# Development of a Thin Film Magnetic Moment Reference Material

**DOI:** 10.6028/jres.113.002

**Published:** 2008-02-01

**Authors:** D. P. Pappas, S. T. Halloran, R. R. Owings, F. C. S. da Silva

**Affiliations:** National Institute of Standards and Technology, Boulder CO 80305

**Keywords:** AGM, magnetic reference material, magnetic thin film, magnetometer, SQUID, VSM

## Abstract

In this paper we present the development of a magnetic moment reference material for low moment magnetic samples. We first conducted an inter-laboratory comparison to determine the most useful sample dimensions and magnetic properties for common instruments such as vibrating sample magnetometers (VSM), SQUIDs, and alternating gradient field magnetometers. The samples were fabricated and then measured using a vibrating sample magnetometer. Their magnetic moments were calibrated by tracing back to the NIST YIG sphere, SRM 2853.

## 1. Introduction

Magnetic thin film materials are used extensively in magnetic media, recording heads, low field sensors, and isolators. Applications often require films with thicknesses from 1 to 20 nm. Accurate measurement of the magnetic moment and thickness of these films is challenging because of their shape and low moment. A discussion of the importance of thin film standards for these types of samples was presented in our earlier work [1] in the context of an interlaboratory comparison (ILC). In the present work, we refined the shapes and calibrated the total magnetic moments of a set of thin film, ferromagnetic samples against a bulk magnetic standard reference material (SRM). The final result is a set of low moment, low coercivity samples that can be used as reference materials in instruments such as vibrating sample magnetometers (VSMs), SQUIDs, and alternating gradient field magnetometers.

The ferromagnetic SRMs that NIST presently produces are bulk samples, including a Ni sphere, SRM 772a (2.383 mm diameter); an yttrium iron garnet (YIG) sphere, SRM 2853 (1 mm diameter, 2.8 mg); and a Ni disk, SRM 762 (6 mm diameter, 0.13 mm thick). These SRMs have magnetic moments specified at a field of about 400 kA/m (5000 Oe). The moments of the two Ni SRMs are on the order of 10^−3^ A·m^2^ (1 emu), whereas the YIG sphere total moment at saturation is about 76 μA·m^2^ (7.6 × 10^−2^ emu). Due to the high fields and relatively high moments of these samples, it is necessary to change the instrument sensitivity and applied field scales when calibrating for a measurement of a typical thin film sample (on the order of 200 times lower moment).

The motivation for the current work comes from the fact that NIST presently does not supply a ferromagnetic thin film SRM. This leads to calibration of instruments against standards that have significantly different geometry and a moment that can be up to three orders of magnitude higher than the device under test. The goal of this work is to transfer the moment measured from the SRM 2853 to a new reference material that has magnetic properties and dimensions that more closely represent typical samples measured in industry, i.e., lower moment, a square hysteresis loop with low coercivity, and thin film geometry.

The samples in this work were fabricated to answer the industry and scientific community needs based on feedback from our previous work [1] and a second ILC (see Appendix A). Another important result of these studies was that, while several different magnetometers were used, we found that the vibrating sample magnetometers had sufficient reproducibility to characterize these samples, providing that we could calibrate these instruments with sufficient accuracy. This indicates that low moment, ferromagnetic reference materials would have a high impact. Below, we present the calibration and characterization of our VSM instrument as well as the fabrication and measurement of the magnetic properties of the samples. These samples are currently being considered as a candidate for a low moment reference material (RM 8140).

## 2. Development of Thin-Film Magnetic Reference Material

### 2.1 Sample Fabrication

The samples were developed at NIST based on informal feedback from magnetometer manufacturers and industrial users who had participated in the two ILC’s (Ref. [1] and Appendix A). These prototype samples were fabricated at NIST with a standard bilayer liftoff process to define the pattern for the magnetic material. Optical lithography allowed the lateral dimensions of the films to be determined with an expanded uncertainty, coverage factor *k* = 2 [2], of about 1 μm. This estimate is based on manufacturer specifications of the pattern generator, stepper, and visual inspection during the processing. A high purity Si(100) wafer, 75 mm in diameter and 400 μm thick, was used as a substrate with an amorphous, 150 nm thick, thermal oxide layer grown on the wafer prior to depositing the magnetic material. The magnetic layer, consisting of 5 nm Ta/100 nm Ni_81_Fe_19_/5 nm Ta, was then sputter deposited onto the patterned wafer. The wafer was subsequently diced into 76 coupons that measure 5 mm × 6 mm with magnetic films that were 2 mm × 4 mm.

**Fig. 1:**
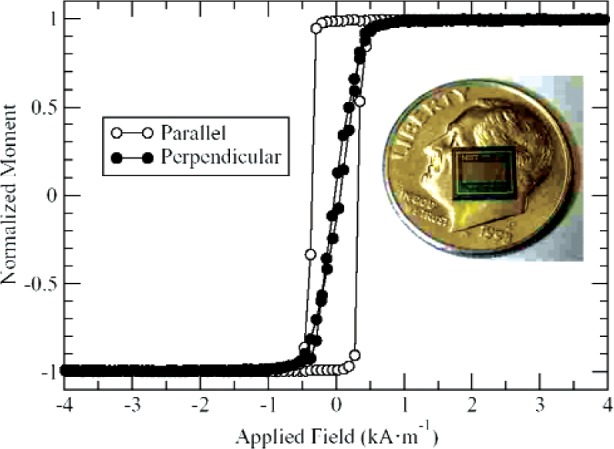
VSM data illustrating the uniaxial anisotropy of the magnetic reference sample, shown in inset. Data was taken with field parallel to the long axis of the rectangular film and perpendicular.

The most significant difference between these samples and the ones described in the second ILC (see Appendix A) is that they have about twice the magnetic moment. In addition, the shape was changed from a 4 mm × 4 mm square to a 2 mm × 4 mm rectangle to make alignment of the sample in the magnetometer easier. The films were grown in a magnetic field of 15.9 kA/m (200 Oe) oriented along the long axis of the rectangle in order to induce a uniaxial anisotropy [3]. VSM hysteresis loops measured with the magnetic field applied parallel and perpendicular to the long axis of the sample, as well as a picture of the die, are shown in Fig. 1. The existence of a uniaxial anisotropy in the direction of the long axis is seen in both the squareness of the easy axis loop and in the low remanence of the hard axis loop below the saturation field [4].

### 2.2 Calibration Against SRM 2853

Two aspects are important when determining the properties of the reference material: (1) the Type B uncertainty [2] of the calibration value of the SRM traceable to a fundamental standard SI unit, and (2) the Type A statistical uncertainty of the measurements (i.e., the deviations from measurement to measurement calculated from the standard deviation of the measured quantities). For the rest of this paper, all uncertainties will be quoted for a coverage factor (standard deviation) of 1, i.e., *k* = 1 from Ref. [2], unless otherwise specified.

In particular, the absolute accuracy is evaluated by calibrating against the NIST SRM 2853. The VSM provides a measured voltage for both the NIST SRM 2853 and the die being measured, and the total moment of the die is calculated using:

$${\text{moment}}_{\text{Die}}={\text{Voltage}}_{\text{Die}}\times \left(\frac{{\text{mass}}_{\text{SRM}}}{{\text{Voltage}}_{\text{SRM}}}\times {\left[\frac{\text{moment}}{\text{mass}}\right]}_{\text{SRM}}\right),$$ (1)

where Voltage_SRM_ and Voltage_Die_ represent the measured voltages of the SRM 2853 and the unknown selected die. The SRM moment/mass is given in the NIST certificate as (27.6 ± 0.1) A·m^2^/kg (*k* = 2) [5]. All measurements in this paper were conducted at room temperature of (295 ± 2) C. This is within the temperature range given in Ref. [5], so no temperature correction was made to this moment. The mass of the SRM was determined using a calibrated microbalance, and was measured to be (2.752 ± 0.0005) mg using a 5 digit, NIST traceable scale. A field of 400,000 A/m was applied during our measurement of the SRM. The reproducibility of the SRM moment measurement was determined by measuring it independently five times. We accomplished this by removing and remounting the SRM each time. From these measurements we obtained Voltage_SRM_ = (363 ± 3) μV. In Eq. (1), the results inside of the parentheses are a conversion factor to take the voltage measured on a die to the total moment. With the measured mass, voltage, and the given moment/mass, we obtain a conversion factor of (0.209 ± 0.002) A·m^2^/V for our VSM system, where the dominant uncertainty (0.9 %) in this result comes from voltage measurement.

Because the moments of the SRM 2853 and the thin films samples used in this study differ by two orders of magnitude, the 500 μV and the 5 μV scales on the VSM lock-in amplifier were used. Therefore, it was necessary to determine the linearity of the instrument between these two ranges. This was accomplished by injecting a 100 Hz signal into a resistor divider network and measuring it with both a NIST traceable voltmeter and the lock-in amplifier. The divider contained three resistors in series, R1, R2, and R3 (values shown in caption of Table 1). For the lock-in calibration, the signal was injected across the entire network, and the voltages on the two scales were measured across R2+R3 and R3. For the voltmeter, the signal was injected across R2+R3, and measured at R3. Table 1 shows the test voltages and ratio results. For our system, the ratio of the voltages across the resistor bridge measured with the calibrated voltmeter compared to the ratio measured by the lock-in amplifier deviated by less than 0.1 % on the same scales used for the standard and sample measurements.

One other potential problem for calibration is the difference in the masses of the SRM and the measured film. The SRM has a mass of about 2.7 mg while the reference material samples have a mass of about 27 mg. However, the mass of the VSM rod that vibrates is on the order of 200 g, i.e., more than 4 orders of magnitude higher than the difference in the sample masses. In addition, it is locked in a feedback loop to maintain the modulation frequency and amplitude. Therefore, we conclude that the mass differences between the sample and the SRM are negligible from the perspective of the VSM measurement.

### 2.3 Sample Measurement

**Fig. 2:**
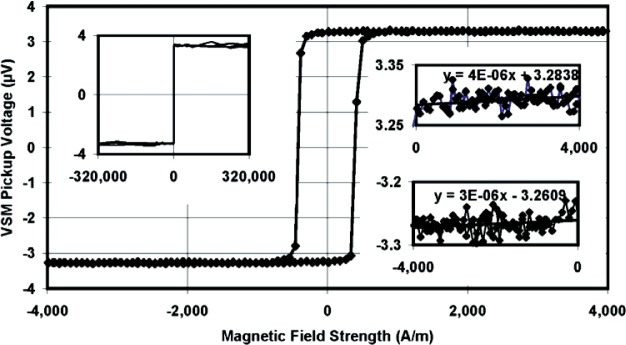
An uncalibrated voltage signal hysteresis curve of die 16 from reference wafer number 1 illustrates the saturation moment uncertainty. The saturation moment is the average of the absolute value of the points in the inset plots.

A hysteresis curve from the VSM of one of the samples is shown in Fig. 2. The low scatter in the saturation moment is illustrated in the inset plots for the saturation regions. This shows that the signal from the sample can be measured very accurately in the VSM. In addition, contributions from the silicon substrate were negligible at low field (4 kA/m, i.e., about 50 Oe). This is confirmed by measuring the sample at a very high field, as shown in the left inset of Fig. 2, where the field was ramped to 320 kA/m (~4000 Oe). The absence of diamagnetic contributions is evident because the slope of the saturated branches of the hysteresis curve is effectively zero even at these very high fields. Finally, as shown in Bozorth [6], the temperature dependence of the Ni_81_Fe_19_ is very close (and most likely smaller) to that of pure Ni. From the NIST Ni sphere and disk reference material standards, SRM 772a and SRM 662 [7], we find that the uncertainties due to temperature are negligible in the room temperature range that the samples were measured, T = (295 ± 2) K.

**Fig. 3:**
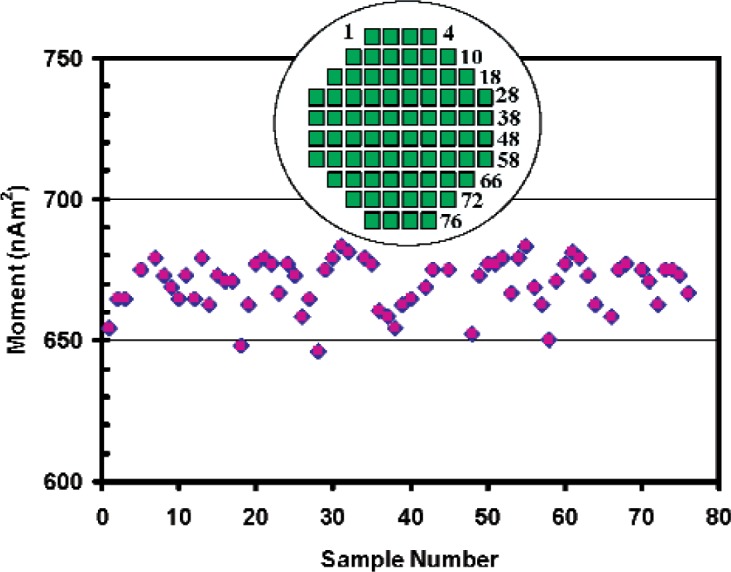
Survey data of entire wafer. Inset shows the physical positions of the dies on the wafer.

In order to evaluate the homogeneity of the film thickness across the wafer and the reliability of the VSM measurement, a full survey of the moments of the samples was undertaken. The results are shown in Table 2 and Fig. 3, where the calibrated moment is tabulated and plotted as a function of the sample number on the wafer. The physical position of the dies on the wafer is shown in the inset. These data show that there is a systematic periodicity of the sample moments from the right side of the wafer to the left, with the measured moments all between 646 – 683 nA·m^2^. This shows that the homogeneity across the wafer is about 5.7 % and that the VSM measurement is capable of resolving this level of variation. This is expected from the results of the ILC presented in Appendix A.

When reporting a moment for a NIST SRM or RM, the numerical average value of the lot is reported. In addition, it is necessary to combine the Type A measurement uncertainty from Table 2, 1.3 %, with the standard uncertainty of the conversion factor of 0.9 %. Therefore, we find the nominal average value for the sample moments to be:

$$M_{s}=(670\pm 11)\text{nA}\cdot m^{2}(k=1).$$

### 2.4 Magnetometer Considerations

When using a flux-measurement technique such as a VSM to compare a sample to a reference material, it is necessary to ensure that the measurement is conducted in the dipole regime for both samples. In other words, we need to confirm that differences in sample shape do not affect the measurement. In this section we consider these effects and show that for our VSM these conditions hold.

**Fig. 4:**
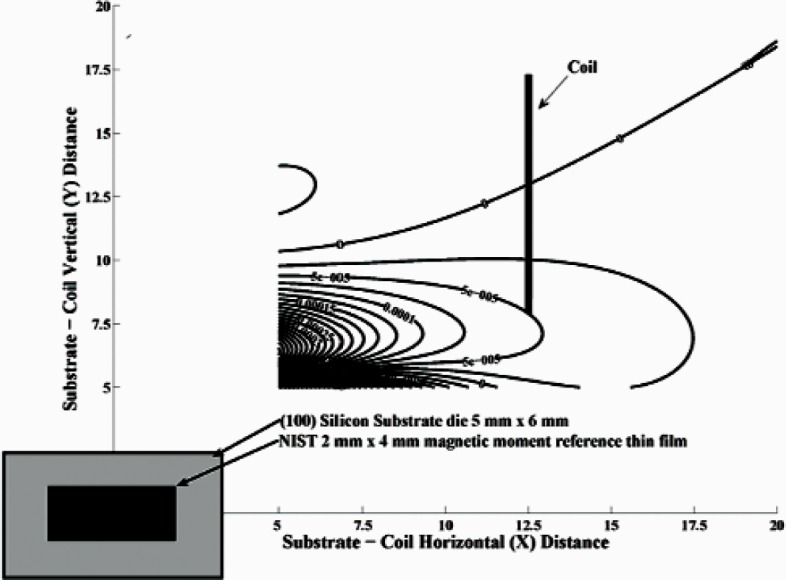
VSM sample-coil diagram and calculated magnetic field plot for NIST VSM. The plotted contour shows the ratio of the quadrupole contribution to the magnetic field signal divided by the dipole contribution as a function of position (in mm) from the center of the sample.

Our measurements were conducted on a relatively common VSM configuration, specifically with four 2.5 cm diameter differential pickup coils situated in the gap of an electromagnet on a 2.5 cm × 2.5 cm square and centered on the sample position. This geometry is illustrated in Fig. 4 for one quadrant, with the sample shown to-scale at the center. Because our samples were rectangular, the calculation of the dipole and quadrupole moments was performed using a multi-pole expansion [8]. The contour plot of the ratio of the quadrupole to the dipole contributions of the magnetic field as a function of position from the center of the sample is depicted in Fig. 4. From this figure we can see that the quadrupole moment is roughly two orders of magnitude smaller than the dipole moment for each position calculated.

This shows that the rectangular shape of the reference sample does not have a significant quadrupole contribution to the overall detected moment over the coil area. Therefore, the shape difference is not a significant factor for measurement of these samples in this VSM. However, it is important to note that not all VSM instruments are configured identically. Moment measurements are strongly dependent on the sample size relative to the pickup coil dimensions. It is therefore incumbent upon the user to ensure that the sample size is appropriate with respect to the pickup coil and vibration amplitude to ensure an accurate calibration and reproducible moment measurement [9].

## 3. Conclusions

Low-moment, low-coercivity, thin film samples that have a square hysteresis were developed as reference materials for magnetometers. Results from a second inter-laboratory comparison of a new generation of low magnetic moment thin film samples were presented. From these investigations, the sample shape and magnetic properties were chosen to have the greatest possible impact on magnetometers such as SQUIDs, alternating gradient field magnetometers, and vibrating sample magnetometers. The average saturation moment was measured to be of (670 ± 11) nA·m^2^ (*k* = 1) at room temperature, T = (295 ± 2) K. These samples should satisfy a need in the scientific community for a ferromagnetic, thin film reference sample for calibration of magnetometers when measuring low moment samples at low fields.

## Figures and Tables

**Fig. 5:**
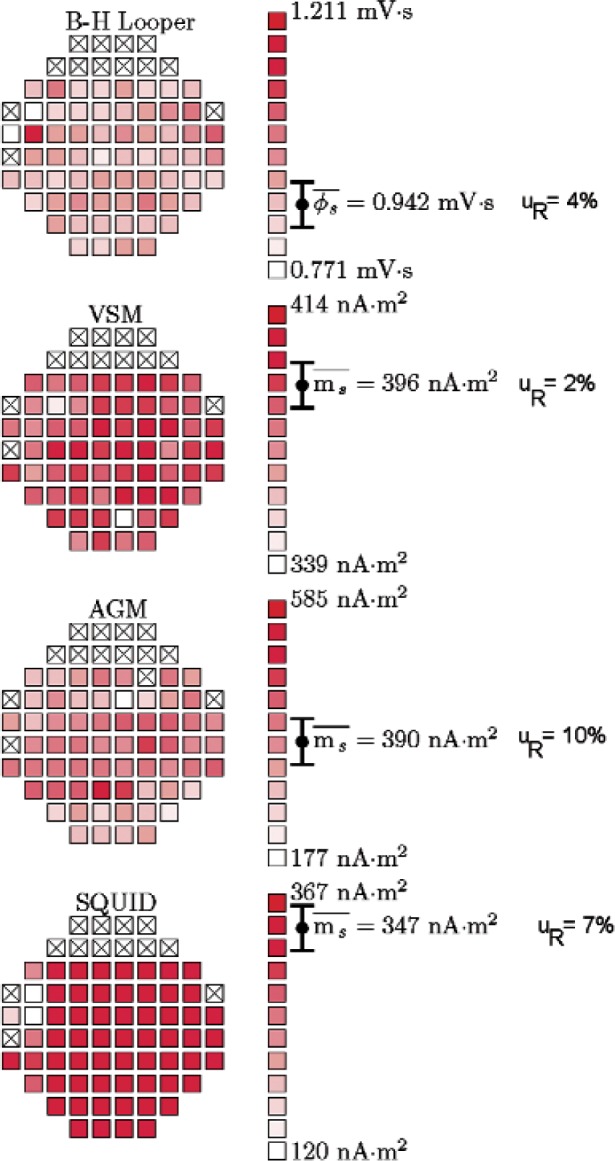
NIST measurements of the saturation magnetic moment *m*_s_ and flux Φ_s_ across the same wafer for the four different measurement techniques. The scale on the right indicates the grayscale range of moment values. The error bars correspond to one standard deviation. Samples marked with an “x” were not used in this study due to visual defects. Values reported for AGM and SQUID rely on manufacturer’s calibration. VSM values were calibrated against the NIST Ni sphere, SRM 772a. BH-Looper results are not calibrated.

**Table 1:** Calibration voltages for 100 Hz signal of the resistor bridge and the lock-in amplifier with a NIST traceable voltmeter (serial number 86860115 by ISO certification No. U0018, calibrated July 19, 2004, report #1610376-86860115). The resistor bridge consisted of three resistors in series: R_1_ = 9.96 MΩ, R_2_ = 989 Ω, R_3_ = 33.2 Ω.

	Test 1	Test 2	Test 3
Lock-in Amplifier			
Supply Voltage (V_R1+R2+R3_)	0.955	2.861	4.767
V_R2+R3_	102.7·10^−6^	308.2·10^−6^	513.5·10^−6^
V_R3_	3.3·10^−6^	9.995·10^−6^	16.6·10^−6^
V_R3_/V_R2+R3_	32.13·10^−3^	32.43·10^−3^	32.32·10^−3^
Voltmeter			
Supply Voltage (V_R2+R3_)	0.955	2.861	4.767
V_R3_	30.89·10^−3^	92.66·10^−3^	154.3·10^−3^
V_R3_/V_R2+R3_	32.34·10^−3^	32.38·10^−3^	32.37·10^−3^
Voltmeter/Lock-in Ratio	1.007	0.9985	1.001

**Table 2:** Full survey of the reference material wafer. The samples are numbered according to their position on the wafer, as shown in Fig. 3.

Sample	M	Sample	M	Sample	M
#	nA·m^2^	#	nA·m^2^	#	nA·m^2^
1	654	26	658	54	679
2	665	27	665	55	683
3	665	28	646	56	669
5	675	29	675	57	663
7	679	30	679	58	650
8	673	31	683	59	671
9	669	32	681	60	677
10	665	34	679	61	681
11	673	35	677	62	679
12	665	36	660	63	673
13	679	37	658	64	663
14	663	38	654	66	658
15	673	39	663	67	675
16	671	40	665	68	677
17	671	42	669	70	675
18	648	43	675	71	671
19	663	45	675	72	663
20	677	48	652	73	675
21	679	49	673	74	675
22	677	50	677	75	673
23	667	51	677	76	667
24	677	52	679	Average	670
25	673	53	667	Std. Dev.	9.0(1.3%)

**Table 3:** Magnetic moments (measured by NIST) of the 14 pairs of ILC samples that were measured by the various laboratories. The units for *m*_s_ are nA·m^2^ for the VSM, AGM, and SQUID, and mV·s for the Looper.

Die #	Sample A		*u*_M_	*u*_R_	Die #	Sample B		*u*_M_	*u*_R_	Ratio A/B	
	Inst.	*m*_s_				Inst.	*m*_s_			m^a^_s_/m^b^_s_	*u*_N_
15	VSM	414	2	20	61	VSM	391	2	6	1.06	0.05
29	VSM	383	2	5	36	VSM	397	2	23	0.96	0.06
	AGM	354	5	138		AGM	428	2	65	0.83	0.2
17	SQUID	354	1	–	58	SQUID	306	–	1	1.16	–
22	VSM	400	2	4	47	VSM	410	2	7	0.98	0.02
34	VSM	412	2	10	55	VSM	401	3	3	1.03	0.02
71	SQUID	357	1	–	76	SQUID	350	–	1	1.02	–
30	VSM	404	2	15	51	VSM	405	2	3	1.00	0.04
25	VSM	396	4	7	26	VSM	396	2	12	1.00	0.04
16	VSM	387	2	8	37	VSM	402	2	12	0.96	0.04
32	Looper	941	9	94	49	Looper	930	9	93	1.01	0.14
33	VSM	407	2	26	46	VSM	404	2	7	1.01	0.07
	SQUID	353	1	–		SQUID	362	1	–	0.98	–
10	VSM	393	2	3	70	VSM	393	2	20	1.00	0.05
42	VSM	402	2	6	53	VSM	404	2	7	1.00	0.02
11	Looper	1050	11	105	35	Looper	922	9	92	1.14	0.14

**Table 4:** Saturation moment/flux measurement ratios of the laboratory comparison wafer performed by the partner laboratories. Measurements made by laboratory #9 on samples 25 and 26 were rejected on statistical analysis basis for outliers [10]. The units for *m*_s_ are nA·m^2^ for the VSM, AGM, and SQUID, and mV·s for the Looper.

Lab #	Sample A			Sample B			Ratio A/B
	Die #	Inst.	*m*_s_	Die #	Inst.	*m*_s_	*m*^a^_s_/*m*^b^_s_
1	15	VSM	378	61	VSM	378	1.00
2	29	VSM	356	36	VSM	365	0.98
		AGM	352		AGM	353	1.00
3	17	SQUID	382	58	SQUID	356	1.07
4	22	VSM	367	47	VSM	362	1.01
5	34	VSM	357	55	VSM	353	1.01
7	71	SQUID	360	76	SQUID	352	1.02
8	30	VSM	352	51	VSM	359	0.98
9	25	VSM	490	26	VSM	610	0.80
11	16	VSM	376	37	VSM	374	1.01
13	32	Looper	65	49	Looper	55	1.18
14	33	VSM	375	46	VSM	352	1.07
		SQUID	374		SQUID	361	1.04
16	10	VSM	359	70	VSM	371	0.97
17	42	VSM	426	53	VSM	423	1.01
19	11	Looper	32.2	35	Looper	34.7	0.93

**Table 5:** Reported magnetic moments from VSM measurement results for samples in ILC for NIST and partner laboratories. Units for *m*_s_ are nA·m^2^. The uncertainty, *u*, was calculated as the standard deviation of the moment averages.

	Average Moment		Ratio	
	*m*_s_	*u*	*m*^a^_s_/*m*^b^_s_	*u*
NIST	401	14 (3%)	1.00	0.04
Partner Laboratories	371	21 (6%)	1.00	0.03

## 4. Appendix A. – Inter-laboratory Comparison

The inter-laboratory comparison (ILC) to determine optimal sample properties was an important step in the development of the reference material samples. In the study we enlisted the voluntary participation of laboratories at magnetometer manufacturers, and industrial, academic, and government institutions. Each laboratory measured two nominally identical magnetic samples (i.e., same size and shape from the same wafer) and reported the measured moments. Measurement techniques used by the participants included a traditional vibrating sample magnetometer (VSM), alternating gradient magnetometer, superconducting quantum interference device magnetometer, and inductive looper magnetometer.

The laboratories were asked to report both the moments of the samples they measured. We then compiled the data and compared the absolute moments as well as the ratio of the two measurements at each laboratory. A 6 % variation in the absolute moments was found, however, only a 3 % variation in the measured ratios was observed. This indicates that a reference sample would have a significant impact in order to bring inter-laboratory measurements into agreement. In this Appendix, we describe these measurements in detail.

For this second inter-laboratory comparison, 76 samples were produced from a single wafer. Each sample was a square Ni_81_Fe_19_ (Permalloy) film with dimensions of 4 mm × 4 mm × 20 nm on a silicon substrate. The magnetic properties of the samples, namely saturation magnetic moment, *m*_s_, and flux, Φ_s_, were measured at NIST using four different techniques, using methods discussed in our previous work [1]. These include vibrating sample magnetometry (VSM), alternating-field gradient magnetometry (AGM), superconducting quantum interference device (SQUID) magnetometry, and inductive magnetometry (B-H looper).

The twenty partner laboratories each were assigned a confidential identification number and shipped a pair of randomly selected dies from the wafer for analysis. Each laboratory received procedural instructions and a form for the measured quantities. The form to be filled out by the operator included the relevant saturation quantity (moment or flux) value, uncertainty, the units for each of the two samples, and two questions regarding the importance of this type of sample.

From our previously published ILC results we found that uncertainty arising from discrepancies in absolute quantities is minimized when comparing two sample moments, as in a regular calibration procedure [1]. Therefore, in this study we distributed two samples to each lab. This allows us to more easily evaluate the efficacy of these samples as magnetic moment references. Also, because the samples were from the same wafer, the ratio of the saturation moments (m^b^_s_/m^a^_s_) was expected to be close to one.

Initial measurements on the samples (i.e., dies) were made at NIST with all four magnetometry techniques. Figure 5 shows the moment (in grayscale) and position of each die across the wafer for each technique. The averages were calculated from the 63 good dies, and the percentage deviation is shown for each technique as u_R_. All the techniques showed random uncertainties, but the VSM had the lowest, at 2 %.

Twenty random pairs of samples were then selected from the wafer to be distributed to each of the original partner laboratories. Of these labs, results were obtained from fourteen. Therefore, we restrict our analysis to the 28 samples that were measured by responding labs. Before the samples were sent out, another round of measurements was conducted on them at NIST. The results of these measurements are shown in Table 3. Included in this table are the moments and relevant measurement uncertainties. Specifically, three types of uncertainties reported in this table. The measurement uncertainty (*u*_M_) [10], is the intrinsic uncertainty of the instrument without removing the sample. This represents, for example, the amplifier noise and reproducibility of measurement when the magnetic field is cycled. The reproducibility uncertainty (*u*_R_) is defined as one standard deviation among the averages from each of three repeated measurements on a given sample. For this measurement, the sample is removed and re-mounted each time. Finally, the net uncertainty of the reference test ratio, *u*_N_, is calculated by combining in quadrature the larger uncertainties (in this case *u*_R_) for the two dies.

Turning to the results of the ILC, all of the 14 responding laboratories reported measurements of the absolute moment and ratios. Ten of the labs used the VSM technique, allowing for a statistically significant analysis. However, too few laboratories with SQUID magnetometers, B-H loopers, or AGMs replied for meaningful analysis. These facts, combined with the low uncertainties for the VSM shown in Figure 5, lead us to focus on the VSM as a candidate for the reference sample.

Looking back at the NIST VSM measurements of Table 3, the uncertainty for repeated measurements on a single sample was only 0.5 %. Therefore, the relatively large uncertainty from die to die (*u*_R_) is the dominant uncertainty in these measurements. Combining the uncertainties of nine good VSM measurements, we find an average value of *u*_R_ = 3 % [10]. The ratio for the sample pairs gives a value of (m^b^_s_/m^a^_s_) 1.00, with a standard uncertainty for these nine measurements of 4 %.

The results of measurements taken by the partner laboratories are shown in Table 4. From the *m_s_* data for the partner laboratories, the inter-laboratory moment measurement gives an uncertainty of 6 %. This is a factor of two higher than the NIST values for the same dies. The uncertainty in the ratio measurements was 3 %, in good agreement with the NIST result. Since no uncertainties for the measurements were reported from the partner laboratories, error propagation from the individual measurements is not included.

Table 5 summarizes the results from NIST and the partner laboratories. This leads to the most important result of the ILC, i.e., that the uncertainty of the VSM ratio measurement for the various partner laboratories is half that of the absolute moment measurement. In addition, the ratio and absolute moment uncertainties for the same dies measured at NIST were very close to each other and to the partner laboratory ratio uncertainty. More specifically, the intra-laboratory data (moment uncertainty for NIST and ratio uncertainty for NIST and partners) show that an achieved accuracy of 3 % is possible if an appropriate reference sample is used correctly. High moment uncertainty of 6 % between partner laboratories illustrates that a reference sample is needed for calibration to bring all labs into agreement. In addition, we note that the data for the moments reported by the NIST VSM measurements and the partner laboratories are inconsistent, i.e., the values do not fall within the uncertainties. This indicates that there is a bias in the measurements that can be attributed to the fact that none of the measurements had been calibrated against a primary, SI traceable standard using a specific calibration method. This shows that a reference material would be of value to bring the absolute moment measured at different laboratories into agreement.

Finally, the participants were interviewed to determine the response to two questions. The first question asked whether the existing and planned NIST standards would meet the participant’s present and future needs. Three of the respondents replied in the negative. However, each felt that the development of a new magnetic thin film standard would have a high impact. The other seven respondents reported satisfaction with the existing standards produced by NIST, but they all indicated that a new standard would benefit general magnetometer users. In general, users of magnetometers were in the first group, while magnetometer manufacturers were in the second. The users felt additional NIST standards would be extremely useful and cost effective because of savings in time and error correction.

The second question addressed the impact of existing standards and a potential thin-film standard for materials characterization. Ten laboratories responded to this question, which included comments from users at industrial labs indicating that they all needed better standards and felt the cost savings would be thousands of dollars each year. In addition, all of the magnetometer manufacturers acknowledged the importance of magnetic thin film standards to their customers.
